# Supplementary material for: A Rapid Quantitative Analysis of Bicomponent Fibers Based on Cross-Sectional In-Situ Observation
Source: Polymers (Basel). 2023 Feb 8;15(4):842. doi: 10.3390/polym15040842 (PMC9964497; doi:10.3390/polym15040842)
Supplement: Supplementary file 1 [file polymers-15-00842-s001.zip › polymers-2188014-supplementary.pdf]

Supplementary Materials

A Rapid Quantitative Analysis of Bicomponent Fibers Based on Cross-Sectional In-Situ Observation

Jieyao Qin [1], Mingxi Lu [1], Bin Li [2], Xiaorui Li [1], Guangming You [1], Linjian Tan [1], Yikui Zhai [3], Meilin Huang [1,*] and Yingzhu Wu [1,*]

[1] School of Textile Materials and Engineering, Wuyi University, Jiangmen 529020, China
[2] College of Innovation and Entrepreneurship, Wuyi University, Jiangmen 529020, China
[3] College of Intelligent Manufacturing, Wuyi University, Jiangmen 529020, China
* Correspondence: hml@wyu.edu.cn (M.H.); wyz@wyu.edu.cn (Y.W.)

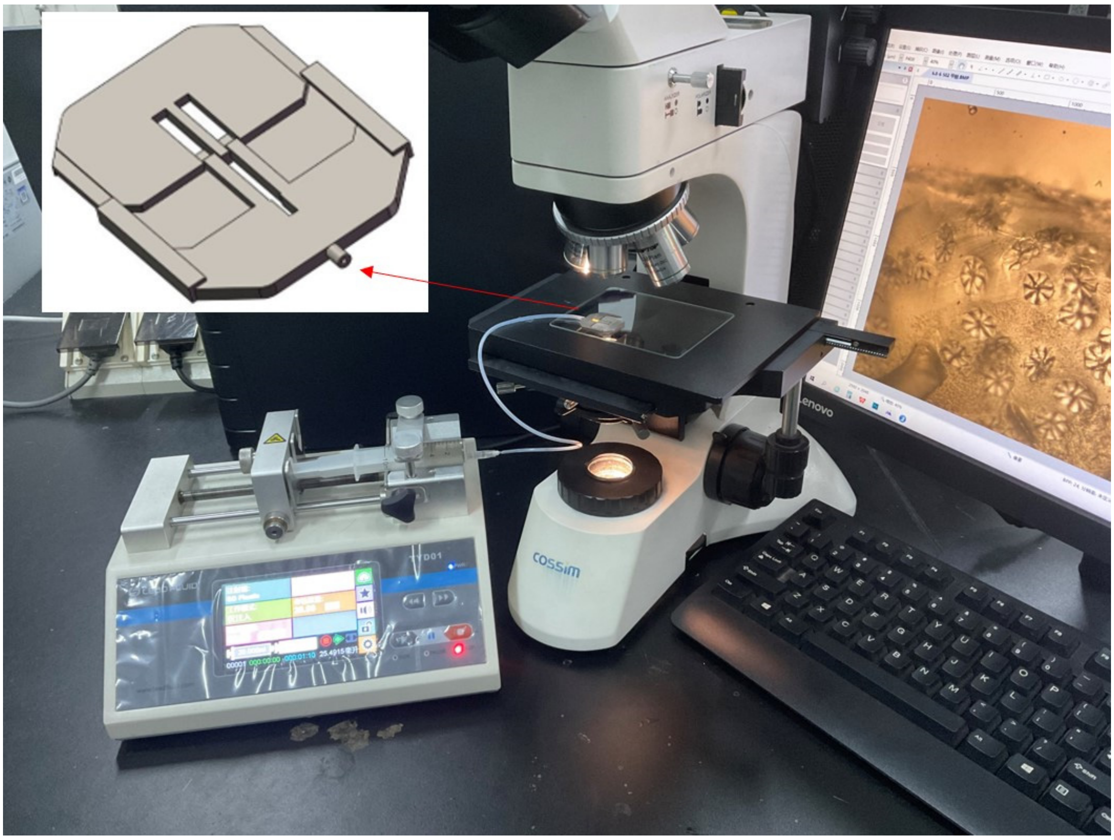

Figure S1. In-situ dissolving equipment and the new slicer.

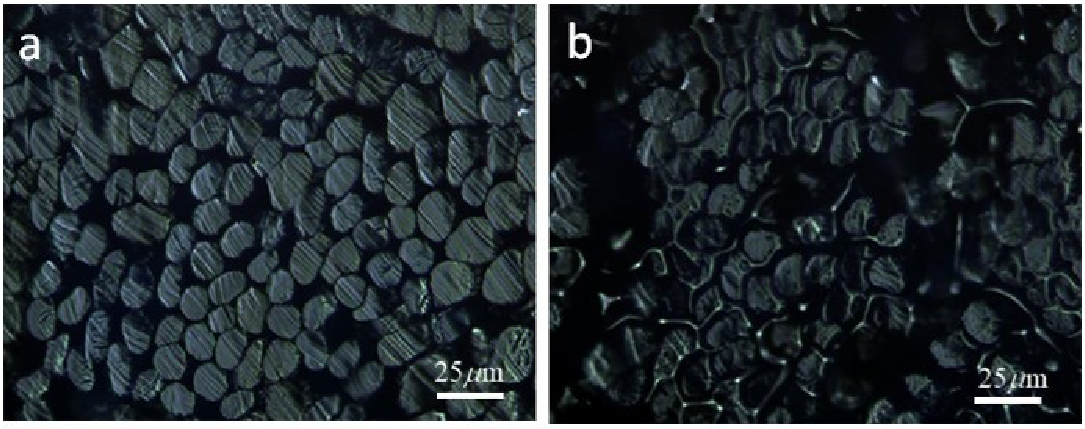

Figure S2. In-situ dissolving of wool/ acrylic fibers(**a**) Before adding basic sodium hypochlorite solvent, (**b**) After adding basic sodium hypochlorite solvent to dissolve the wool fibers.

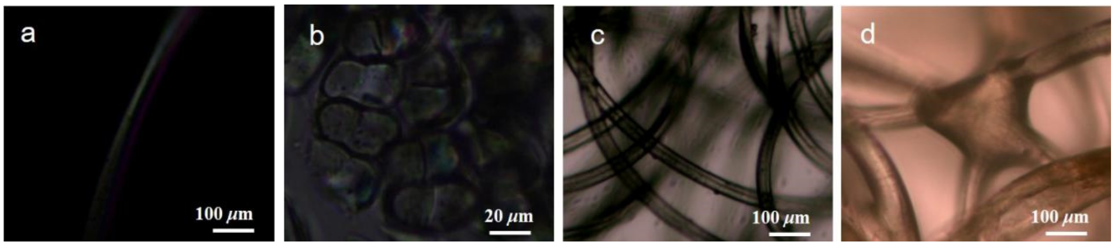

Figure S3. the Melting process of side-by-side bicomponent fibers (PET/PTT) with longitudinal direction(**a**) the polarization performance of PET/PTT bicomponent fibers with longitudinal direction, (**b**) the cross-section of PET/PTT bicomponent fibers, (**c**)Before heated to melt, (**d**) PET/PTT bicomponent fibers with longitudinal direction were heated to melt.

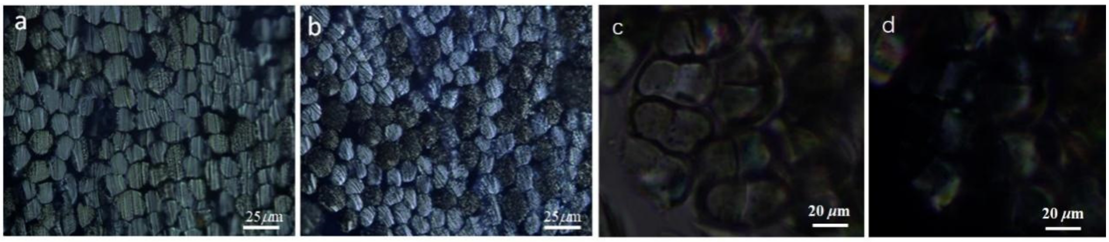

Figure S4. the polarization performance of wool/ acrylic blended fibers and side-by-side PET/PTT bicomponent fibers (**a**) wool/ acrylic blended fibers before polarizing experiment, (**b**) wool/ acrylic blended fibers after polarizing experiment, (**c**) the cross-section of PET/PTT bicomponent fibers before polarizing experiment, (**d**) the cross-section of PET/PTT bicomponent fibers after polarizing experiment.

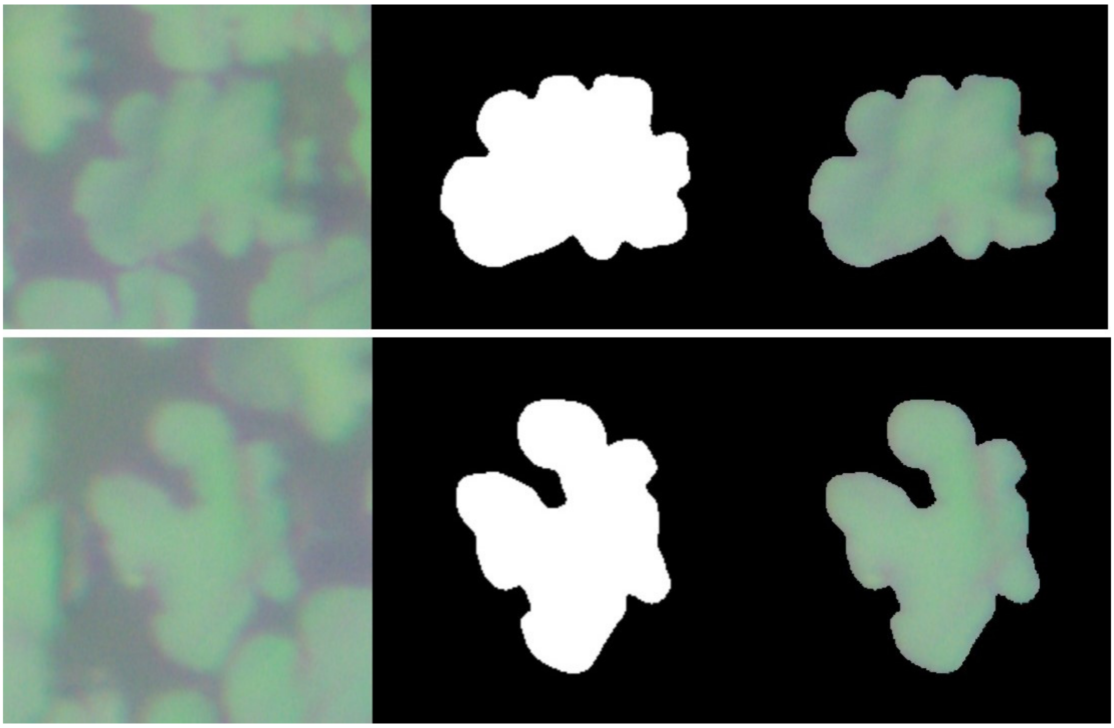

Figure S5. Labeled images of viscose fibers for establishment of their identification model.
